# The Extent and Coverage of Current Knowledge of Connected Health: Systematic Mapping Study

**DOI:** 10.2196/14394

**Published:** 2019-09-25

**Authors:** Maria Karampela, Minna Isomursu, Talya Porat, Christos Maramis, Nicola Mountford, Guido Giunti, Ioanna Chouvarda, Fedor Lehocki

**Affiliations:** 1 IT University of Copenhagen Copenhagen S Denmark; 2 University of Oulu Oulu Finland; 3 Imperial College London London United Kingdom; 4 Aristotle University of Thessaloniki Thessaloniki Greece; 5 Maynooth University Maynooth Ireland; 6 Slovak University of Technology Bratislava Slovakia

**Keywords:** connected health, health services research, interdisciplinary research, empirical research, telemedicine, information technology, wireless technology, health informatics, information systems

## Abstract

**Background:**

This study examines the development of the connected health (CH) research landscape with a view to providing an overview of the existing CH research. The research field of CH has experienced rapid growth coinciding with increasing pressure on health care systems to become more proactive and patient centered.

**Objective:**

This study aimed to assess the extent and coverage of the current body of knowledge in CH. In doing so, we sought to identify specific topics that have drawn the attention of CH researchers and to identify research gaps, in particular those offering opportunities for further interdisciplinary research.

**Methods:**

A systematic mapping study that combined scientific contributions from research in the disciplines of medicine, business, computer science, and engineering was used. Overall, seven classification criteria were used to analyze the papers, including publication source, publication year, research type, empirical type, contribution type, research topic, and the medical condition studied.

**Results:**

The search resulted in 208 papers that were analyzed by a multidisciplinary group of researchers. The results indicated a slow start for CH research but showed a more recent steady upswing since 2013. The majority of papers proposed health care solutions (77/208, 37.0%) or evaluated CH approaches (49/208, 23.5%). Case studies (59/208, 28.3%) and experiments (55/208, 26.4%) were the most popular forms of scientific validation used. Diabetes, cancer, multiple sclerosis, and heart conditions were among the most prevalent medical conditions studied.

**Conclusions:**

We conclude that CH research has become an established field of research that has grown over the last five years. The results of this study indicate a focus on technology-driven research with a strong contribution from medicine, whereas the business aspects of CH have received less research attention.

## Introduction

### Background

A variety of terms and concepts exist that describe the use of technology in health including *Health IT*, *eHealth*, *Telemedicine*, and *Health Informatics*. In the early 2000s [1], the term *Connected Health* (CH) began to appear. It did so in the context of research that investigated how information and communication technologies could advance health by connecting people, knowledge, technical artifacts, and organizations. Some years later, Poon and Zhang introduced a new CH information system based on a “four-layered architecture: personal, home, community, and hospital” [2]. One of the most cited definitions for CH was introduced in 2013 [3]:

Connected Health encompasses terms such as wireless, digital, electronic, mobile, and tele-health and refers to a conceptual model for health management where devices, services or interventions are designed around the patient’s needs, and health related data is shared, in such a way that the patient can receive care in the most proactive and efficient manner possible. All stakeholders in the process are connected by means of timely sharing and presentation of accurate and pertinent information regarding patient status through smarter use of data, devices, communication platforms, and people.

The European Network for the Joint Evaluation of Connected Health Technologies defines the CH vision as “a paradigm shift looking after the individual and community health in a process that speaks to the health journey of the person, through the entire lifespan, leveraging a variety of technologies to do so” [4]. Achieving this vision will require attention to be paid to policy and regulation, technology and interoperability, training and education, business and revenue models, as well as citizen and clinician engagement [5]. Successful implementation of the CH vision needs time and effort from all health care stakeholders.

### Objectives

Given the recent growth in CH research, we set out to map its evolution up to the present day. We use a systematic mapping study to chart the research landscape combining scientific contributions from the research disciplines of medicine, business, computer science, and engineering. Although previous CH reviews do exist, they are confined to descriptions of how CH solutions are being used in specific conditions such as cancer [6,7] or in specific measurement technologies such as measurement of vital signs [8] or weight [9]. Past efforts also concentrated on presenting CH-related literature in systematic ways [6,10,11]. Our goal is to provide a comprehensive, interdisciplinary overview of existing CH research. This will help researchers understand how the field has developed since its earliest studies in the late 1990s. It will also identify those topics that have drawn the attention of the research community. This knowledge will add value by identifying gaps or interdisciplinary opportunities in the study of CH. Perhaps most importantly, it may also underpin future work to develop an integrated and interdisciplinary research agenda for CH that will answer efficacy, design, policy, and sustainability questions for patients, clinicians, technology developers, and businesses.

## Methods

### Overview

**Figure 1:**
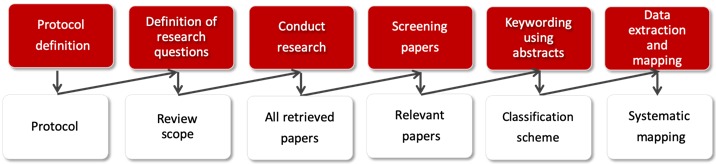
Systematic mapping process.

This paper followed a systematic mapping study method [12]. Systematic mapping studies aim primarily to present an overview of a research area to report the quantity and type of literature and results that are published within it. The systematic mapping process comprises 3 steps: (1) the identification of relevant literature, (2) the composition of a classification scheme, and (3) the mapping of literature [12]. Figure 1 presents the mapping process including the search for relevant literature, the definition of a scheme, and the mapping of relevant publications.

The method was used to examine the body of existing research conducted by researchers in medicine, business, computer science, and engineering to understand the nature of research conducted in the area of CH. A systematic mapping study was found to be suitable for this task as it provided a high-level framework for combining interdisciplinary research efforts as well as an analytical framework that spanned disciplinary boundaries.

### Mapping Questions

The aim of this study was to present an overview of the available publications pertinent to CH. Following the systematic mapping study method, the study was guided by a set of mapping questions. Table 1 presents the 6 mapping questions (MQs) and the rationale for conducting this study. More detail as to the logic supporting the selection of suitable MQs is included in the Data Extraction Strategy section below. The study search strategy as well as the inclusion and exclusion criteria (EC) were based on these 6 MQs.

**Table 1:** Mapping questions.

ID	Questions	Rationale
MQ^a^1	Which publication channels are the main targets for CH^b^ research?	To identify where CH research can be found and to identify targets for publication of future studies
MQ2	How has the frequency of studies related to CH changed over time?	To identify the publication trends over time of CH literature
MQ3	What are the research types of CH studies?	To explore the different types of research reported in the literature concerning CH
MQ4	Are CH studies empirically validated?	To discover whether research on CH has been validated through empirical studies
MQ5	What are the approaches that were reported in CH research?	To discover the CH approaches reported in the existing CH literature
MQ6	What are the main topics and conditions in CH literature?	To identify the research areas and health conditions discussed in papers

^a^MQ: mapping question.

^b^CH: connected health.

### Search Strategy

As CH is inherently interdisciplinary, our goal was to use the systematic mapping method to study research contributions on the topic across disciplines. We, therefore, searched papers from the most recognized scientific literature databases in each of the chosen disciplines.

The papers of the study were retrieved from 7 databases of scientific literature, namely, (1) Institute of Electrical and Electronics Engineers Xplore Digital Library, (2) Association for Computing Machinery Digital Library, (3) ScienceDirect, (4) SpringerLink, (5) MEDLINE and PubMed, (6) Business Source Complete (EBSCO), and (7) ABI and INFORM Collection (ProQuest), with the help of the corresponding search engines. The search was performed in October 2018. Different search strings were proposed and discussed over the course of joint meetings to arrive at a set of primary keywords. Two of the authors tested different strings of potential keywords such as “Connect Health,” “Connecting Health,” “Connect-Health,” or “Connecting-Health.” After evaluating the search results, the authors agreed to proceed using the following search strings: (“Connected” AND “Health”) OR (“Connected” AND “-” AND “Health”).

The search was applied to the title, abstract, and keywords to include relevant papers. On the basis of our methodology, we included a wide selection of papers on the first iteration and thereafter relied upon the inclusion criteria and EC to identify the relevant literature [13].

### Paper Selection Criteria

The search results that were retrieved from the 7 chosen search engines were merged in a single list and saved in a spreadsheet document. Duplicate entries were removed based on the digital object identifier (DOI) and the intertitle lexical distance (the Levenshtein metric was used to measure the lexical distance) with the help of a custom Python script [14]. A total of 7 papers had to be manually reviewed as potential duplicates.

The final list of papers was distributed to all authors for analysis. On the basis of the area of expertise, pairs of authors were assigned to analyze papers retrieved from each database. A total of 3 areas of expertise were identified as follows: technical (computer science and engineering), medical, and business. Each pair of authors reviewed the title, abstract, and keywords and made a recommendation as to whether that paper should be included or excluded. Discrepancies across coding teams were resolved through further scrutiny of the paper.

**Figure 2:**
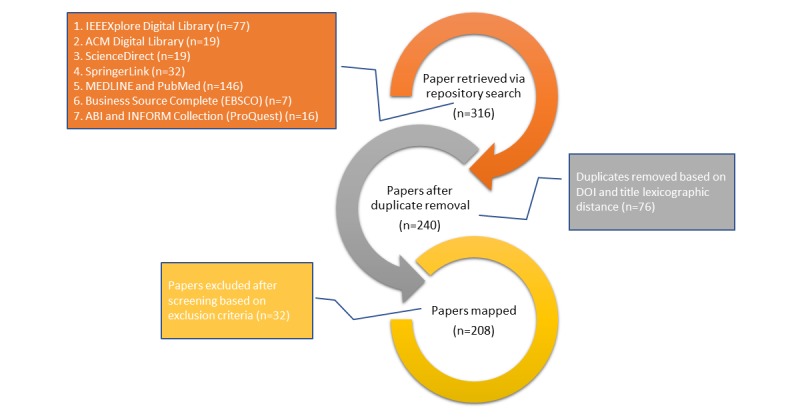
Study selection process. ACM: Association for Computing Machinery; DB: database; DOI: digital object identifier; IEEE: Institute of Electrical and Electronics Engineers.

The inclusion criteria were limited to studies that discussed CH. In total, 240 papers were identified after the removal of duplicates, whereas 32 papers were excluded after meeting at least one of the following exclusion criteria: EC1—papers that focus on CH comorbidities, that is, the paper discusses the connection between health conditions, not CH as a concept; EC2—papers that focus on law; EC3—papers that focus on medical procedures only, without connection to CH; and EC4—papers that focus on evaluating climate but not in relation to CH. Figure 2 presents the process of the study selection.

### Data Extraction Strategy

The selected studies were analyzed to collect information that would give answers to the MQs according to the data extraction strategy outlined in Table 2 below.

**Table 2:** Data extraction strategy.

Mapping questions	Description of classification categories
MQ^a^1	Publication source and publication channel
MQ2	Publication year
MQ3	Research types [15]: evaluation research—real-world CH^b^ approaches are implemented and undergo evaluation; solution proposal—a new CH solution or a significant extension of an existing solution is proposed, and the evaluation of the solution is based either on empirical data or theoretical argumentation; opinion paper—a CH study that is based on the personal opinion of the author(s); review—studies that present a review of existing CH literature; other—the remainder of research types associated with CH studies. This is assigned to studies where research type is either unknown or does not fall into one of the aforementioned main categories (eg, experience papers, which express the personal experience of author(s) without providing any scientific evidence to support it)
MQ4	Empirical types [16,11]: case study—an empirical inquiry that investigates a CH approach within its real-life context; survey—an empirical inquiry method for collecting quantitative information concerning a CH approach, for example, a questionnaire; experiment—an empirical method applied under controlled conditions to evaluate a CH approach; history-based evaluation—nonempirical studies evaluating CH approaches in previously completed projects; theory—nonempirical research approaches or theoretical evaluation of a CH approach; other—the remainder of CH studies that do not fit within the previous types
MQ5	Contribution types: method—a manner of procedure and steps taken to acquire knowledge in CH; tool-based technique—a technique based on a software tool to accomplish CH tasks; model—a system representation that allows CH to be investigated through a hierarchical structure; framework—a real or conceptual structure intended to serve as a support or guide for CH; other—the remainder of CH approaches. This includes CH studies of approach not fitting other classes, along with very rare approaches that have been grouped in this category to facilitate abstraction and visualization. The approaches grouped herein are feasibility study, field research, process, guideline, and network analysis
MQ6	Main topics and medical conditions

^a^MQ: mapping question.

^b^CH: connected health.

MQ6 comprised 2 parameters: the topic of the paper and the medical condition examined within it. With regard to the topic of the paper, we did not have a predefined list of topics but relied on an open coding process where the researchers conducting the analysis selected a descriptive word for the topic of the paper. To identify the main topics of the included papers, the authors relied on the title, abstract, and keywords. After compiling all topic words, we curated the topic list to come up with a consistent list (eg, using the same word for *body-worn sensors* and *wearables*). Similarly, with regard to the medical condition examined, no predefined classification was used that allowed coders to assign the medical condition, if any, of each CH paper. Again, the resulting condition list was curated to develop a more consistent list (eg, using the same word for *ageing* and *elderly*).

### Synthesis Method

The synthesis method used was based on the following steps: (1) enumerating the number of papers per publication channel and the number of papers per bibliographic source per year; (2) enumerating the primary studies that are classified in each MQ’s response; (3) presenting visualizations for the classification results, which have been used in the analysis; and (4) presenting a narrative summary to discuss the principal findings.

## Results

### Overview

**Figure 3:**
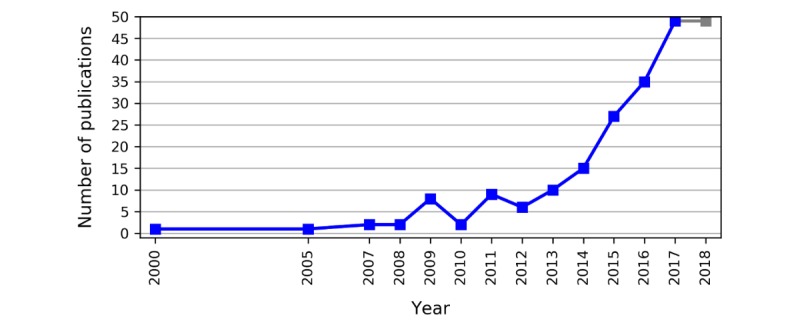
Publication trend per year−total number of connected health papers published per year; the number of papers reported for the year 2018 only includes papers published until October 2018, with a projection of estimated papers based on linear extrapolation (presented as superimposed gray line).

**Figure 4:**
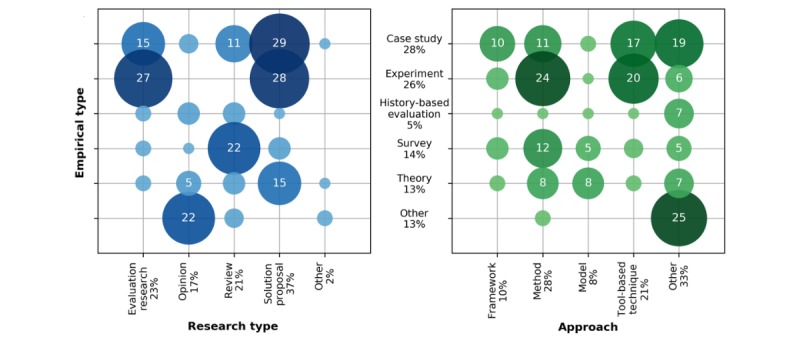
Bubble graphs associating the empirical types with the research types (left) and the approaches (right) of the included connected health studies. The vertical axis (empirical type) is shared between the two graphs. The size and shade of each bubble represents the absolute frequency of connected health papers belonging to a given pair of empirical type and research type (left) or approach (right); absolute frequencies less than 5 are not typed inside the bubble because of space limitations. The horizontal and vertical axes labels are accompanied by the relative frequency (ie, percentage) of the class.

This section describes the results related to the systematic MQs presented in Table 1. Multimedia Appendix 1 gives an overview of the classification results for all the included papers [6-11,17-217]. Custom Python scripts have been developed to process the classification data and generate the tables (Multimedia Appendix 1) and figures (Figures 3 and 4) of this section. The Pandas and the Matplotlib Python [218,219] libraries were used to manipulate the tabular input data and plot the results, respectively.

### Mapping Question 1: Which Publication Channels are the Main Targets for Connected Health Research?

The majority of the CH papers were published in scientific journals (139/208, 66.8%), whereas 32.9% (68/208) were published in scientific conferences. Table 3 lists the publication forums that have published at least two CH papers.

### Mapping Question 2: How Has the Frequency of Studies Related to Connected Health Changed Over Time?

Figure 3 presents the publication trend per year from 2000 to 2018. It must be noted that the analysis does not include the full body of CH work published in 2018 as our search was performed before the end of that year. We have added a projection for year 2018, estimating the total number of papers. Our projection assumes that publication frequency would remain consistent till the year end. As Figure 3 shows, there is a gap in publications from 2001 to 2004. Less than 5 papers per year were published until 2008, whereas from 2013 to 2017, the number of publications rises steadily.

### Mapping Question 3: What are the Research Types of Connected Health Studies?

Figure 4 shows the research types of the included studies. The majority of the papers are solution proposal studies (77/208, 37.0%), 23.5% (49/208) of the selected studies evaluated CH approaches, 20.6% (43/208) reviewed literature, 16.8% (35/208) reported the opinions of authors about CH, and 1.9% (4/208) of the papers analyzed were classified as other.

### Mapping Question 4: Are Connected Health Studies Empirically Validated?

Figure 4 presents our findings as to whether or not the included studies were empirically validated and, if so, the empirical validation approaches used. The majority of the studies were, in fact, empirically validated. More specifically, 28.3% (59/208) were validated in case studies, 26.4% (55/208) in controlled experiments, and 13.4% (28/208) with surveys. Nonempirical papers included, for example, those focused on theory (25/208, 12.0%) and those using history-based evaluation (11/208, 5.2%).

### Mapping Question 5: What Are the Approaches That Were Reported in Connected Health Research?

Figure 4 shows the wide range of approaches taken in the included CH studies. The approaches most frequently reported belonged to other categories (66/208, 31.7%), followed by methods (56/208, 26.9%) and tool-based techniques (43/208, 20.6%). Only 10.0% (21/208) of the papers were classified as presenting frameworks, with 8.1% (17/208) suggesting models.

### Mapping Question 6: What Are the Main Topics and Diseases in Connected Health Literature?

Tables 4 and 5 present the results for the main topic and condition of the included CH studies. Topics with fewer than two occurrences have been omitted from Table 4. The most common research topics of the included CH papers were health care (×16) and disease management (×13), followed by telemedicine/telehealth (×11) and electronic health (eHealth)/mobile health (×9). Table 4 lists all research topics and their frequencies, whereas Table 5 lists the conditions and their frequencies. As for the main conditions reported in our study, diabetes (×12), aging (×10), cardiovascular diseases (×10), cancer (×7), chronic diseases (x7), and dementia (x4) were among the prevalent conditions found in the selected studies.

**Table 3:** Publication sources that have published 2 or more connected health papers.

Publication source	References	Number of published papers
Studies in Health Technology and Informatics	Topaz and Pruinelli (2017) [37], Wu et al (2017) [44], O’Neill et al (2012) [73], Feied et al (2009) [81], Goossen (2017) [82], Singh and Kvedar (2009) [84], Maglaveras et al (2016) [85], Saranto et al (2017) [96], Skiba et al (2016) [101], Kreuzthaler et al (2017) [106], Goossen-Baremans et al (2017) [107], Sermeus et al (2016) [110], Silvello (2018) [206], Tonheim and Babic (2018) [207]	14
Journal of Medical Systems	Wen (2013) [26], Das and Goswami (2013) [27], Santos et al (2016) [28], Chang et al (2013) [29], Vlahu-Gjorgievska et al (2016) [57] Xu and Wu (2015) [59], Lin et al (2016) [65], Xie et al (2014) [80], Kim and Lee (2014) [97]	9
Telemedicine and eHealth^a^	Ford et al (2018) [31], McConnochie et al (2016) [41], Kvedar et al (2009) [49], Aberger et al (2014) [72], Trout et al (2017) [90], Ternullo et al (2013) [99], Kvedar et al (2009) [122]	7
JMIR mHealth and uHealth	Wang et al (2018) [21], El Amrani et al (2017) [51], Dur et al (2018) [62], Wang et al (2018) [64], Harte et al (2017) [77], Argent et al (2018) [100], Sathyanarayana et al (2016) [108]	7
Health Affairs (Millwood)	Frist (2014) [48], Kvedar et al (2014) [52], Iglehart (2014) [55], Weinstein and Lopez (2014) [75]	4
JMIR^b^	Sperrin et al (2016) [9], Gay and Leijdekkers (2015) [39], Loiselle and Ahmed (2017) [89], Agboola et al (2015) [115]	4
QJM^c^: An International Journal of Medicine	Caulfield (2013) [88], Ansary et al (2013) [114], Agboola et al (2013) [116], Caulfield and Donnelly (2013) [3]	4
Journal of Diabetes Science and Technology	Watson et al (2008) [38], Watson et al (2009) [63], Pelletier et al (2011) [78], Helal et al (2009) [109]	4
Journal of the American Academy of Audiology	Saunders and Jacobs (2015) [36], Krupinski (2015) [83], Gladden et al (2015) [112]	3
JMIR Research Protocols	Mountford et al (2018) [32], Wang et al (2018) [58]	2
International Journal of Medical Informatics	Giunti et al (2018) [6], Karampela et al (2018) [11]	2
Maturitas	Chouvarda et al (2015) [17], Stara et al (2018) [68]	2
JMIR Human Factors	Harte et al (2017) [22], Harte et al (2018) [71]	2
Journal of Personalized Medicine	Agboola and Kvedar (2012) [7], Jethwani et al (2010) [35]	2
Revue de l'infirmiere	Warnet (2017) [18], Raymond and Léo (2017) [19]	2
In the 31st International Symposium on computer-based medical systems	Barbosa et al (2018) [138], Barbosa et al (2018) [189]	2
In the 14th International Conference on Telecommunications	Starič et al (2017) [135], Maramis et al (2017) [204]	2
In Proceedings of the International Workshop on Software Engineering in Healthcare Systems	Carroll and Richardson (2016) [172], Abdullah et al (2018) [183]	2
BMC^d^ Medical Informatics and Decision Making	Allaert et al (2017) [118], Tharmalingam et al (2016) [119]	2
In the First International Conference on Connected Health: Applications, Systems, and Engineering Technologies	Sinharay et al (2016) [132], Gawanmeh (2016) [194]	2
Journal of Evaluation in Clinical Practice	Barr et al (2012) [50], Barr et al (2014) [103]	2
American Journal of Hospice and Palliative Medicine	Aktas et al (2015) [54], Thomas et al (2017) [111]	2

^a^eHealth: electronic health.

^b^JMIR: Journal of Medical Internet Research.

^c^QJM: Quarterly Journal of Medicine.

^d^BMC: BioMed Central.

**Table 4:** Frequencies of the main topics associated with the reviewed articles; main topics with a single occurrence have not been included because of space limitations (N=208).

Topics	Frequency (%)
Health care	16 (7.6)
Disease management	13 (6.2)
Telemedicine/telehealth	11 (5.2)
Electronic health/mobile health	9 (4.3)
Monitoring	8 (3.8)
Security	8 (3.8)
Consumer health informatics	7 (3.3)
Sensors	6 (2.8)
Information and communication technologies challenges	5 (2.4)
Personal health devices	4 (1.9)
Education	3 (1.4)
Usability	3 (1.4)
Medical education	3 (1.4)
Innovation	3 (1.4)
User-centered design	3 (1.4)
Interoperability	3 (1.4)
The internet of things	3 (1.4)
Software engineering	2 (0.9)
Privacy	2 (0.9)
Medication adherence	2 (0.9)
Lifestyle coaching	2 (0.9)
Ageing	2 (0.9)
Personalization	2 (0.9)
Service delivery	2 (0.9)
Elderly	2 (0.9)

**Table 5:** Frequencies of the target conditions associated with the reviewed articles; of note, only 77 of the reviewed articles have been mapped to a target condition (N=208).

Condition	Frequency (%)
No (condition)	131 (62.9)
Diabetes	12 (5.7)
Ageing	10 (4.8)
Cardiovascular diseases	10 (4.8)
Cancer	7 (3.3)
Chronic diseases	7 (3.3)
Dementia	4 (1.9)
Multiple sclerosis	3 (1.4)
Stroke	2 (0.9)
Mental health	2 (0.9)
General health	2 (0.9)
Psychosis	1 (0.4)
Chronic skin disease (psoriasis, dermatology)	1 (0.4)
Vital signs	1 (0.4)
Stress	1 (0.4)
Renal conditions	1 (0.4)
Malaria	1 (0.4)
Hemodialysis	1 (0.4)
Dental issues	1 (0.4)
Arrhythmia	1 (0.4)
Obesity	1 (0.4)
Palliative care	1 (0.4)
Urinary incontinence	1 (0.4)
Epilepsy	1 (0.4)
Rheumatoid arthritis	1 (0.4)
Glaucoma	1 (0.4)
Blood transfusion service	1 (0.4)
Hearing issues	1 (0.4)
Environmental exposure	1 (0.4)

## Discussion

This section discusses the results and main findings of this study. First, each mapping question is discussed in its specific subsection. Finally, the limitations of this study are discussed.

### Principal Findings

#### Publication Channels

Publication channels provide information about how research activities in CH have been established in the scientific community. The results of this study show that the majority of CH publications appear in peer-reviewed scientific journals (139/208, 66.8%). Although both journals and conferences aim to disseminate research and contribute to the development of a field, journals are typically considered more prestigious because of manuscript review criteria and acceptance practices. In addition, journals usually present more extended pieces of research work and contribute toward the establishment of a knowledge base for a field. Although the process of publication is longer compared with conferences, journals potentially have a larger impact in terms of visibility and audience reach [220-222]. CH studies are often published in peer-reviewed journals with high impact factors, such as the *Journal of Medical Systems*, *Studies in Health Technology and Informatics*, *Telemedicine and eHealth*, and *Journal of Medical Internet Research*. This indicates that CH research efforts to date have focused on the establishment of a body of knowledge. On the other hand, conferences offer a dynamic environment that enables researchers to communicate with colleagues, exchange research interests, and receive specialized feedback. Conference publications could, therefore, be considered innovation-laden venues that denote the evolution of research in a field, as they often facilitate the presentation of novel ideas based on preliminary results. Conferences appear to be channels for the introduction and establishment of innovative research to experts of a particular area of research [220-222]. The first published conference papers on the topic of CH appeared in 2016. In the following two years, CH research was accepted and published by two international conferences. This finding could indicate efforts to further develop a research community to highlight and nurture CH research. The establishment of the European Network for the Joint Evaluation of Connected Health Technologies research coordination program, the funding of two Innovative Training Network CH projects (cancer: activating technology for connected health [CATCH] and connected health early stage researcher support system [CHESS]), and the expansion of the European Connected Health Alliance organization across Europe have all served to connect stakeholders under the umbrella of CH [223-225]. This coordination of effort has potentially led to the increase in both journal and conference publications as researchers come together around a shared interest in CH.

#### Publication Trend

The papers matching our inclusion criteria, while spanning a publishing period from 2000 to 2018, show that CH research has attracted increasing attention since 2013. A publication gap between 2001 and 2004 coincides with the infancy of the CH field, likely reflecting the fact that the evolution of CH research was a slow and gradual process underpinned by the fermentation of experts from different academic disciplines. Overall, Figure 3 draws a very typical picture of the evolution of a research field, with sporadic publications in the beginning followed by an exponential increase in the yearly production of scientific literature. One could argue that the discourse around personal health records (PHRs) and patient services started around early 2000s, whereas the focus until that point had tended toward classic telemedicine. The PHR concept, and the shift from *telemedicine* to patient-centered services may have then driven the publication of CH-related papers. The growth of publications after 2009 can be attributed to various factors. The emergence of CH in the past decade coincides with a demographic shift where the older population is ultimately projected to outnumber the young people [226]. This increasing aging population with its chronic and degenerative diseases has been projected to exert severe financial pressure on future health care systems [226,227]. At the same time, the development of new technologies has facilitated the promotion of CH solutions. The proliferation of devices and apps enabled by *internet of things* in health care over the last decade [228], along with the adoption of smartphones and wearables by everyday users, has transformed health care delivery, enabling remote health monitoring and personalization of health care services [17,229,230]. Likewise, providers such as Amazon, Google, Salesforce, International Business Machines corporation, and Microsoft began to establish new data centers for hosting cloud computing apps in 2009 [231]. Taking that into consideration, we could argue that the emergence and evolution of the CH approach from 2009 onward reflects a demand for the provision of CH services to exploit technological advances and bring together patients and stakeholders to “offer the correct information to the correct person at the correct time” and make better decisions for health and care [17]. In terms of eHealth policies and regulations, the trends are consistent with the development of the CH paradigm as, according to the World Health Organization, the number of countries with eHealth and telehealth policies or strategies has started to increase significantly since 2009 [232].

#### Research Types

Our results show that CH researchers focused primarily on suggesting novel solutions or extending existing research to explain, identify, and provide details of the CH approach (77/208, 37.0%). The 23.5% (49/208) of papers that centered on evaluation also represent attempts to comprehend and develop previous research through evaluating a solution with a valid approach [13,15]. This leads us to conclude that much of CH research is at a development stage, where new concepts are proposed, developed, and evaluated to demonstrate their potential value. However, the existence of a significant number of literature reviews (43/208, 20.6%) indicates that there is maturity in the discipline that allows reviews of the existing research. Through these reviews, researchers aim to identify the research gaps to drive the growth of future research endeavors. If we view this finding alongside the publication trend, which shows that the body of CH research grew over the last few years, then we could argue that there is rapid growth in this discipline. This argument is given weight by the commission of European Union–funded projects such as CHESS in September 2015 and CATCH in 2016 [223,224]. Another factor to consider is that CH as a vision builds upon the best possible utilization of health data. Therefore, the growth of CH research can be considered to also be related to the availability of relevant health data. From 2016 onward, the amount of available networked data is more than 10.000 billion GB, almost double the amount of available data in 2014 [233].

#### Empirical Studies

Our findings support the idea that over half of the CH studies are based on empirically informed approaches. More specifically, the majority of the solution proposals were empirically validated with case studies (59/208, 28.3%), followed by experiments (55/208, 26.4%), whereas 13.4% (28/208) used surveys. Case studies have been shown to be particularly suited to *how* and *why* questions, real-life contexts, and the building of theory [234]. They offer an opportunity to use real-time methodologies where the collection of data and empirical material “takes place at the same time as such data are unfolding and where events depend on each other in a sequential order” [235]. In particular, case studies can offer an “opportunity to observe and analyze a phenomenon previously inaccessible to scientific investigation” [236]. This predominance of case study approaches to the study of CH reflects the relative immaturity of this field of research and a need to build understanding and theoretical contributions in the area through better understanding of individual cases and their contextual parameters. An experiment, on the other hand, is predicated on the analysis of covariance and assumes that participants can be assigned at random, that there are equal numbers of cases in each cell of the factorial design, and “ the correlations between or among the independent variables of a factorial design are zero” [237]. The high number of experiment-based studies can be likely explained by the high number of papers exploring specific technical solutions that can be validated in controlled conditions. This raises the possibility that CH researchers are channeled into empirical approaches that have long been considered *gold standard* in engineering and health research.

#### Approaches

The results for this MQ show that the majority of the included papers belong to other types of studies (66/208, 31.7%), which means that they used approaches that were not defined by our classification scheme. This finding could arise from a variety of factors. More than half of the included papers were from the medical discipline, a fact that has given rise to classification challenges, as reviewers were not able to fit them into one of the defined categories. Although mapping studies are common in the medical discipline [12], classifying research approaches that spanned studies from different disciplines was difficult. A more detailed classification scheme that incorporated classification systems from all disciplines may have delivered more precise results but would have made aggregation difficult and may have obscured any similarities across disciplines. Methods (56/208, 26.9%) were reported to be among the most frequent approaches, followed by tool-based techniques (43/208, 20.6%). This potentially reflects both the relative immaturity and the inherently interdisciplinary nature of the field of CH at this stage. Before researchers can develop models or frameworks that might be applied to CH, they must come to an agreement as to those methods and techniques that are both feasible and acceptable across the new field. The interdisciplinary nature of CH research makes it even more important to focus on methods. As Klein puts it: interdisciplinarity is “a means of solving problems and answering questions that cannot be satisfactorily addressed using single methods or approaches” [238]. Researchers prefer, however, those methods that are traditional to their discipline [239] and so time must be spent discussing and agreeing to those methods and techniques that can span the interdisciplinary boundaries of CH.

#### Research Topics and Conditions

The topic analysis shows that technical and medical disciplines dominate the research topics of the papers, with some references to related disciplines such as education or innovation research. The topic descriptions show a large body of papers discussing measurement-based monitoring with sensors and wearables. This reflects the fast development of body-worn sensors and wireless communication methods that allow the transfer and storage of large amounts of data for further analysis. Another body of papers focused on patient perspectives in CH solutions through a consumer viewpoint or user-centered design, which might reflect an increasing interest in patient empowerment and self-management solutions. The growth of CH as a research field might also reflect regulatory moves toward a data economy where rules for using personal data are clearer (eg, General Data Protection Regulation). Related topics included security, privacy, and interoperability issues. It has been suggested that leveraging interoperable CH technologies for chronic disease management can have multiple positive effects not only on patients but also on clinical outcomes, thus contributing, for example, to the promotion of outpatient care [87]. Although the implementation of CH interoperable scenarios in real-life contexts, such as the Whole System Demonstrator Program in the United Kingdom, had overall positive outcomes [87], issues related to security and lack of data standardization are among the challenges yet to be overcome [106,203].

Diabetes, cancer, and chronic heart conditions dominate the medical conditions covered. This is unsurprising given that these are leading health problems on the global stage [240]. This may also indicate that lifestyle-related conditions are especially suited to CH, as lifestyle changes require patient empowerment and may benefit from technologies used for unobtrusive measurement and personal health devices. Papers discussing issues related to aging, including dementia and falls were often present; among less common conditions, multiple sclerosis seemed to draw more attention.

### Limitations

Owing to the interdisciplinary nature of our topic, we used an interdisciplinary team of researchers for analysis. Having researchers with different backgrounds could decrease inter-rater reliability, especially where we did not have a predefined list of values, as with the *topic* and *condition* parameters. Nevertheless, for the analysis of results, the authors relied upon the interpretation of descriptive statistics and visualizations, thus decreasing the threat to validity. In the same vein, to alleviate the authors’ influence on the classification process, the development of the classification scheme relied on widely accepted guidelines [13]. The differences between content and style of abstracts in different research disciplines and traditions may have resulted in slight differences in information retrieval process. In some cases, the abstract did not include all information needed to classify the paper, and the researchers had to read parts of the full text to obtain all relevant information. However, the vast majority of the 208 publications identified have been classified purely based on the title, abstract, and keywords. In our view, subtle differences in the publication screening process have had only minor impacts on the main conclusions drawn from the 208 publications identified in our study.

The differences in publication practices between the disciplines probably had an influence on how the results of MQ1 were interpreted, as the role of conference and journal publications differs between disciplines [241]. For example, many highly regarded conferences within the business discipline do not publish conference proceedings (eg, European Group for Organizational Studies). Indeed, even those conferences that do publish proceedings may only do so for a subsection of the best papers, and authors will still be offered the option of removing their papers from those proceedings to protect future publishing opportunities (eg, Academy of Management). These factors combine to mean that verbal discussions may well have commenced within the business academic community in conferences that are not reflected within our review as it deals only with published material.

As the methodology of the systematic mapping study that we used in this research was originally developed in the context of software engineering, it is likely that some of the analysis parameters were less optimal in other disciplines. This is reflected, for example, in the large number of studies classified into the category *other* for the parameter *approach*. In the joint analysis meetings, the coding authors shared experiences of their difficulties in classifying the papers within the agreed analysis parameters. Alternative solutions were discussed, but it was difficult to reach a consensus that would have been satisfactory across disciplines. Ultimately, we decided to use the classification parameters proposed by our methodology. The validity of our conclusions is only applicable within the CH context.

To limit the threat related to the identification of primary studies and to include as many relevant papers as possible, 2 of the authors ran several iterations to test different strings of keywords. The adoption of the final set of keywords was used as it returned the largest number of studies. However, the list of studies might be incomplete, as additional or different terms might have an impact on the final selection of papers [242]. Nevertheless, in light of the interdisciplinary endeavor and the scope of the study, we believe that we have included the majority of the relevant literature. For the bibliographic search, no timeframe was defined; hence, the representativeness of the included studies was not affected by this factor. The results of this study should be considered under the prism of the specific search string and classification scheme and offer a baseline for future endeavors.

Our search strategy and inclusion criteria have omitted studies that are referenced in grey literature. However, the literature search was conducted in the world’s most leading and comprehensive databases for scientific knowledge. Furthermore, to alleviate the threat of publications’ nonavailability because of subscription paywalls, we performed the initial screening using a combination of university libraries to improve our access to papers. To address validity threats because of duplication, duplicate entries were removed based on the DOI and the intertitle lexical distance with the help of a custom Python script, which was ultimately manually reviewed to ensure duplicate removal.

### Conclusions

On the basis of our results, we can conclude that CH research is an established field of research. The interdisciplinary nature of the field can be seen especially in papers at the intersection of the medical and technical disciplines. The number of papers in the business research publication forums is still smaller. However, business-related themes are visible in topics of papers, such as consumer orientation and innovation research, although at a much smaller scale than the topics of more technical and medical nature. For CH to succeed, money needs to move differently around the health care system. Most developed health care systems continue to reimburse care in a *cure* rather than a *prevention* mode. Cutting-edge technologies and redesigned care pathways may fail if they run contrary to the flow of health care finance. Our findings emphasize the need to increase business research in the area of CH or to find the vocabularies and keywords necessary to link existing business research with CH endeavors.

There is a growing need to involve and engage patients in their own care and, by extension, in the design of digital solutions to improve their efficacy. Tailored CH interventions may more effectively reach the intended audience in a meaningful way, but this requires in-depth understanding of the condition’s needs, barriers, and facilitators. The important role that health care professionals play in the health care system is in contrast with their lack of involvement in the design of CH. In the same vein, recent research suggests that health care professionals’ education in Europe is lacking in the area of health care information technologies [243]. Emerging trends such as user-centered design and the inclusion of patient representatives attempt to address these problems by creating CH solutions that are tailored to the characteristics and tasks of the intended users. Adoption of these and similar approaches could further the field of CH research and implementation.

Given that CH research has become far more widespread in the years since 2013, perhaps it is time to devote more research resources to the scalability of CH as reflected in empirical approaches that facilitate the use of larger populations.

We see our findings as the foundations of a research roadmap for CH researchers that challenge current thinking in health care. Such a research agenda would go beyond investigations into the feasibility of individual technical solutions to examine and develop ecosystems of stakeholders, technologies, and infrastructures that together form new kinds of systemic solutions. Such an agenda would require more focus on research that addresses interdisciplinary methodological questions alongside the creation of vocabularies and frameworks for researchers working in different disciplines to effectively collaborate and examine interdisciplinary research questions through joint methodological approaches.

## Appendix

Multimedia Appendix 1 Classification results for all the included papers.

## Abbreviations

BMC: BioMed Central
CATCH: cancer: activating technology for connected health
CH: connected health
CHESS: connected health early stage researcher support system
COST: Cooperation in Science and Technology
DOI: digital object identifier
EC: exclusion criteria
eHealth: electronic health
JMIR: Journal of Medical Internet Research
MQ: mapping questions
PHR: personal health record
QJM: Quarterly Journal of Medicine.
